# The effect of Kefir consumption on blood pressure some blood parameters in hypertensive individuals: Erkan Pehlivan

**DOI:** 10.1093/eurpub/ckac131.228

**Published:** 2022-10-25

**Authors:** A Ozyazgan Tokay, E Pehlivan

**Affiliations:** 1 Akcadag Public Hospital, Province Health Directorate, Akcadag/Malatya, Turkey; 2 School of Medicine, Inonu University, Battalgazi/Malatya, Turkey

## Abstract

**Aim:**

In this study is to examine the effect of kefir consumption on blood pressure, some blood parameters and anthropometric measurements in hypertensive individuals.

**Methods:**

The study is a randomized controlled clinical trial. The mean systolic blood pressure in hypertensive individuals was taken as 145.2±19.2. The minimum sample size was determined as 30 for each group with a 95% CI and 80% power. The study was conducted by face-to-face interviews with a total of 100 volunteers, 35 male, 65 female, aged 18 and over, who were hypertensive and volunteered to participate in the study and applied to the internal medicine outpatient clinics of Akçadag Public Hospital between September 2020 and May 2021. The experimental (n = 42) and control (n = 58) groups that the patients are randomly assigned into, the experimental group was ensured to consume 250 ml/day kefir for 28 days. During the study, The study was completed with 56 volunteers. At the beginning of the study and the end of 4 weeks, blood samples, blood pressure, pulse and anthropometric measurements of the individuals were taken.

**Results:**

When the anthropometric measurements were compared, the body weight, body mass index, waist circumference, and hip circumference of the individuals in the experimental group decreased significantly at the end of the study. There was no significant difference between the experimental and control groups in terms of glycemic parameters. While no significant change was observed in the lipid profile in the experimental group, total and LDL cholesterol levels decreased in the control group. There was no statistically significant difference in kidney function parameters in both groups. In addition, at the end of the study, there was no significant change in diastolic blood pressure and pulse in the experimental group, but systolic blood pressure decreased significantly.

**Conclusions:**

Regularly kefir consumption has positive effects on systolic hypertension and weight control.

**Key messages:**

• Regular daily consumption of kefir reduces systolic blood pressure and provides weight control.

• Regular daily consumption of kefir did not lead to an effective result on blood and kidney parameters.

